# IL‐25 promotes cisplatin resistance of lung cancer cells by activating NF‐κB signaling pathway to increase of major vault protein

**DOI:** 10.1002/cam4.2213

**Published:** 2019-05-01

**Authors:** Weiming Shen, Yang Qiu, Jingyao Li, Chao Wu, Zhihui Liu, Xiaorong Zhang, Xiaohong Hu, Yi Liao, Haidong Wang

**Affiliations:** ^1^ Department of Thoracic Surgery Southwest Hospital, Army Medical University (Third Military Medical University) Chongqing China; ^2^ State Key Laboratory of Silkworm Genome Biology, The Institute of Sericulture and Systems Biology Southwest University Chongqing China; ^3^ Vasculocardiology Department Southwest Hospital, Army Medical University (Third Military Medical University) Chongqing China; ^4^ The Institute of Burn Research South‐West Hospital, Army Medical University (Third Military Medical University) Chongqing China

**Keywords:** chemotherapy, cisplatin resistance, IL‐25, lung cancer, MVP

## Abstract

As an inflammatory factor, IL‐25 has been studied in variouscancers, but it is rarely reported in cancer chemotherapy resistance. Major vault protein (MVP), as a gene associated with lung multidrug resistance, is associated with multiple chemotherapy resistances of lung cancer. However, the relationship between IL‐25 and MVP in lung cancer cells has not been studied. In this study, we found that both IL‐25 and MVP were elevated expressed in cisplatin‐resistant lung adenocarcinoma cell line (A549/CDDP). Silencing of IL‐25 resulted in down‐regulation of MVP expression and reduced cisplatin tolerance of A549/CDDP cells. Overexpression of IL‐25 resulted in increase of MVP expression and the cisplatin tolerance in A549 cells. In addition, we found that the extracellular IL‐25 could stimulate the expression of MVP and activate the NF‐κB signaling pathway. Further, animal models also confirmed that IL‐25 reduced the sensitivity of xenografts to chemotherapy. Taken together, we believe that the up‐regulation of IL‐25 induces MVP expression contributing to chemotherapy resistances of lung cancer cells. Our findings suggest that interference the expression of IL‐25 might be potential treatment strategies for the clinical reversing the chemotherapy resistance.

## 1 INTRODUCTION

Lung cancer, especially the nonsmall‐cell lung cancer (NSCLC), is the leading cause of malignant carcinoma‐related deaths.^1^, ^2^ Platinum‐based drugs are currently the first line of chemotherapy for NSCLC, while patients are easily tolerated and tumors are further developed. However, the underlying mechanisms of Platinum‐based drug resistance remain largely unclear.^3^, ^4^, ^5^, ^6^

IL‐25, also called IL‐17E, is a member of IL‐17 family.^7^, ^8^ IL‐25 has a similar structure with IL‐17A, however, its biological function is different from IL‐17A or other IL‐17 family cytokines, such as IL‐17B and IL‐17F.^9^ As the most well‐characterized cytokines in this family, IL‐17A and IL‐17F can induce the recruitment of neutrophils and protect against extracellular pathogens.^9^, ^10^, ^11^ In addition, IL‐17A plays an important role in the development of several autoimmune diseases.^10^ As to IL‐25, it stimulates the production of T helper 2‐type cytokines.^11^ Recent studies have demonstrated that IL‐25 functions as an antitumor factor suppressing tumor growth in several xenograft tumor models, such as melanoma, breast, lung, colon, and pancreatic cancers.^12^, ^13^, ^14^ Contrarily, some studies have also reported IL‐25 may have a tumor promoting effect. For example, increased expression of IL‐17RB (IL‐25 receptor) was strongly correlated with poor prognosis in breast cancer patients, and blocking IL‐17B‐IL‐17RB signaling with an anti‐IL‐17RB antibody resulted in reducing metastasis in pancreatic cancer by silencing NF‐κB‐mediated multiple chemokines release.^15^, ^16^ Therefore, the different functions of IL‐25 in different cells remain to be further revealed.

Major vault protein (MVP), also known as LRP (lung resistance‐related protein), is constitutively expressed in human tissues which participates in the composition of vault particles and nuclear pore complex.^17^, ^18^, ^19^, ^20^, ^21^ Major vault protein (MVP) plays an important role in transporting small molecules from the nucleus to the cytoplasm.^22^, ^23^ Therefore, it is involved in physiological metabolism, self‐protection, anti‐apoptosis, and anti‐infection.^24^, ^25^ Studies have demonstrated that LRP was prevalently expressed in a large set of cancer cell lines which have not been subjected to drug selection, and expressed in certain highly drug‐resistant cell lines without expression of MDRI/Pgp.^26^, ^27^, ^28^ In addition, studies have suggested that increased level of MVP expression indicating a poor response to chemotherapy in many cancers.^29^, ^30^, ^31^ Taken together, these results suggested that multidrug resistance mechanism associated with MVP expression may be widespread in human malignancies. However, the precise molecular mechanism of MVP regulation is largely unknown.

In the present study, we occasionally found that IL‐25 and MVP expressions both increased in cisplatin‐resistant lung cancer cells (A549/CDDP). To further elucidate the relationship of IL‐25 and MVP in drug‐resistant lung cancer cells, we first explored the effect of IL‐25 on A549 and A549/CDDP cells responding to cisplatin treatment. Then, we studied the regulation of IL‐25 on MVP expression in these cells and explored its possible regulatory mechanisms. Finally, in vivo experiments were performed to verify the effect of IL‐25 on chemotherapy response. Our findings elucidated a novel mechanism of IL‐25‐MVP cascade regulated formation of cisplatin resistance.

## 2 MATERIALS AND METHODS

### 2.1 Cell culture and cell lines

Human lung adenocarcinoma A549 cells were cultured in RPMI 1640 medium (SH30809.01, Hyclone, America) containing 10% fetal bovine serum (SV30087.03, Hyclone, America) and 100 μg/mL penicillin/streptomycin (C0222; Beyotim Biotechnology, China) at 37°C with 5% CO_2_. The cisplatin‐resistant A549 cell line (A549/CDDP) was established by treating A549 with gradient increased cisplatin (CDDP, 0.5‐6 μg/mL) for 3 months. Cisplatin (479306‐1G, Sigma‐Aldrich, America) is dissolved with physiological saline solution and protected from light at −20°C.

### 2.2 Cell viability assay and half maximal inhibitory concentration (IC_50_) measurement

Cells were seeded into 96‐well plates (5 × 10^3^ cells/well) and cultured overnight. Then, indicated concentration of CDDP was added to the experimental and control groups, respectively. After incubation for 24 hours, cell counting Kit‐8 reagent (LH650, Dojindo, Japan) was added to each well and incubated at 37°C for 1 hour. The absorbance of each well was measured at 450 nm using a Microplate Reader. The half maximal inhibitory concentration (IC_50_) was measured by linear regression.

### 2.3 Transwell invasion assay

2×10^5^ cells were added into the upper chamber which was precoated with Matrigel (ECM gel, 3422, COSTAR, USA), and cultured in medium without serum. The medium containing 5% fetal bovine serum in the lower chamber served as chemoattractant. After 24 hours of incubation, cells were fixed by precooled methanol for 30 minutes and stained with 0.2% crystal violet solution for 30 minutes. The cells that did not invade through the pores were carefully wiped out with cotton wool. Invasive cells adhering to the undersurface of the filter were counted using an inverted microscope.

### 2.4 ELISA assay

2 × 10^6^ cells were seeded in each well of 6‐well plate and allowed to incubate in CO_2_ incubator at 37°C. Supernatants were collected after 24 hours of incubation. ELISA assay for cytokines IL‐25 was performed using human cytokine ELISA Kit (SEB694Hu, Cloud‐Clone Corp, China) according to manufacturer instructions.

### 2.5 Western blot analysis

Cells in 6‐well plate were allowed to grow to 80% density. For harvesting the protein, cells were washed twice with PBS and lysed with 100 μL ice cold 1× RIPA lysis buffer. For each sample, a total of 50 μg protein was conducted electrophoresis in SDS‐PAGE gel at 100 V for 100 minutes and then transferred to PVDF membrane at 200 mA for 120 minutes by ice‐bath. The membrane was incubated with antibodies of IL‐25 (NBP2‐48858, Novus, USA), MVP (EPR13227(B), Abcam, USA), IκBα (L35A5, CST, USA), phospho‐IκBα (Ser32/36, 5A5, CST, USA), phospho‐NF‐κB p65 (Ser536, E1Z1T, CST, USA), NF‐κB (L8F6, CST, USA) and GAPDH (60004‐1‐Ig, Proteintech, China). ECL kit was used to visualize and analyze the expression of indicated proteins.

### 2.6 siRNA transfection

All small interfering RNAs (siRNAs) used in this study were synthesized by GenePharma (Shanghai, China). HiPerFect Transfection Reagent (Qiagen, Germany) was used to transfect siRNA to A549/CDDP according to manufacturer instructions.

siRNA sequences were listed as follow:

- si‐IL‐25‐#1: 5’‐GGAGAUAUGAGUUGGACAGTT‐3’;
- si‐IL‐25‐#2: 5’‐CUGUCCAACUCAUAUCUCCTT‐3’;
- Negative control siRNA‐1#: 5’‐UUCUCCGAACGUGUCACGUTT‐3’;
- Negative control siRNA‐2#: 5’‐ACGUGACACGUUCGGAGAATT‐3’.

### 2.7 IL‐25 overexpression

IL‐25 coding DNA was amplified from human cDNA library and then sub‐cloned into pCMV3‐C‐GFPSpark plasmid (Sino Biological, China). Empty or IL‐25‐containing plasmid were transfected into A549 cells by using Effectene^®^ Transfection Reagent (Qiagen, Germany) according to manufacturer instructions.

### 2.8 Immunohistochemistry

The streptavidin‐biotin peroxidase complex method was performed for immunohistochemical staining of formalin‐fixed, paraffin embedded tissue sections. Indicated first antibody (1:200) was added for 1 hour at 37°C and 4°C overnight. Sections were permanently developed by using diaminobenzidine (Solarbio, China).

### 2.9 Establishment of xenografts and CDDP treatment

Four‐week‐old female BALB/c nude mice were purchased from the animal center of Army Medical University (Chongqing, China). For in vivo chemosensitivity assays, A549 cells were subcutaneously inoculated into nude mice (8 mice per group, 1 × 10^6^ cells for each mouse). Tumor growth was examined every other day, and tumor volumes were calculated using the equation V = 0.52 × A × B^2^ (mm^3^), A represented the largest diameter and B represented the perpendicular diameter. When the average tumor size reached 500 mm^3^, CDDP was administered by subcutaneous injection at a dose of 5 mg/kg once every 2 days for 3 times in total. The tumor volumes were measured every 2 days for 2 weeks since the last CDDP treatment. Then all mice were sacrificed to harvest the tumor tissues to perform immunohistochemistry assay and hematoxylin & eosin staining. All procedures involving animal were approved by the Committee on the Use and Care on Animals (Army Medical University, Chongqing, China). All animals received humane care according to the criteria outlined in the “Guide for the Care and Use of Laboratory Animals” prepared by the National Academy.

### 2.10 Cytoskeletal staining

Cells (2 × 10^4^) were planted on the carry sheet glass (48‐well size) and allowed to grow to 60% density. Then cells were fixed by 4% paraformaldehyde for 30 minutes. Phalloidine (1:500 diluted, A12379, Thermo Fisher, USA) was used to stain the fixed cells for 1 hour in absence of light. DAPI (C0065, Solarbio, China) was used to stain the nucleus.

### 2.11 Cell apoptosis analysis

After trypsin, cells were collected by centrifuge for 5 minutes at 1000 *g* and washed twice with PBS. Then for each sample, 1 × 10^5^ cells were separated and resuspended into 0.5 mL PBS for apoptosis staining according to manufacturer instructions (C1063, Beyotime, China). Flow cytometry was performed immediately to analyze the apoptosis ratio.

### 2.12 Statistical analysis

Statistical analyses were performed using SPSS.23 software. All data from 3 independent experiments were expressed as mean ± SD. Differences were assessed by two‐tailed Student's *t* test. *P* < 0.01 was considered statistically significant.

## 3 RESULTS

### 3.1 The generation of a cisplatin‐resistant lung cancer cell line

**Figure 1:**
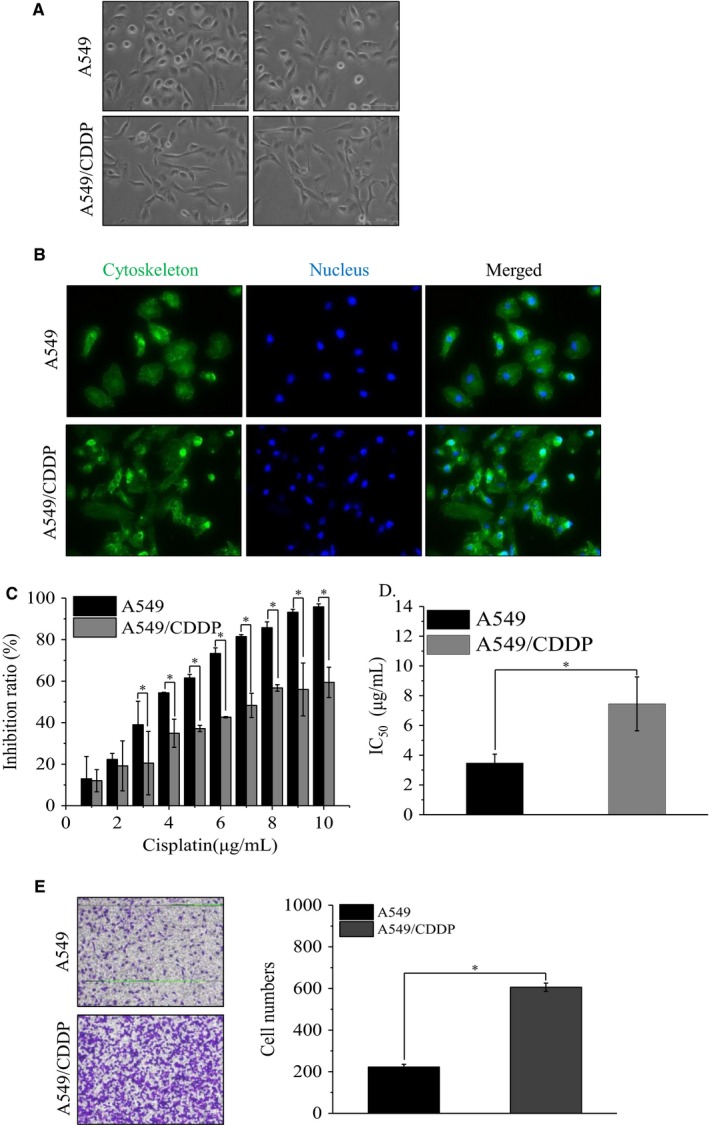
The establishment of a cisplatin‐resistant lung cancer cell line. A, The morphology of parental A549 cells and A549/CDDP cells (magnification, ×100). B, Phalloidine stain the cytoskeleton of parental A549 cells and A549/CDDP cells (magnification, ×200). C, The inhibition ratio of cells treated with different concentration of CDDP and (D) the IC_50_ value of these cells; *P < 0.01 vs A549 cells. Data are means ± SD from three independent experiments. E, The invasion ability of A549 and A549/CDDP cells; *P < 0.01 vs A549 cells. Data are means ± SD from three independent experiments

To better understand the biological mechanisms of chemotherapy resistance in lung cancer cells and reveal the possible reversion opportunities, we generated a cisplatin‐resistant lung cancer cell line (A549/CDDP) by inducing A549 cells with increasing concentration of cisplatin (from 0.5 to 6.0 μg/mL) for 3 months. A549/CDDP cells showed spindle shape and were relatively dispersed (Figure 1A,B). CCK8 cell viability assay results showed that A549/CDDP cells exhibited a stronger cisplatin tolerance (Figure 1C) with the IC_50_ of cisplatin increased 2.15‐folds (Figure 1D), compared to that in parental A549 control cells. Additionally, the invasion ability significantly increased in A549/CDDP cells (Figure 1E), along with the reducing of E‐cadherin and increasing of mmp9 expression (Figure S1A). These data suggested that we have generated a cisplatin‐resistant cell line A549/CDDP.

### 3.2 IL‐25 is associated with cisplatin resistance of lung cancer cells

**Figure 2:**
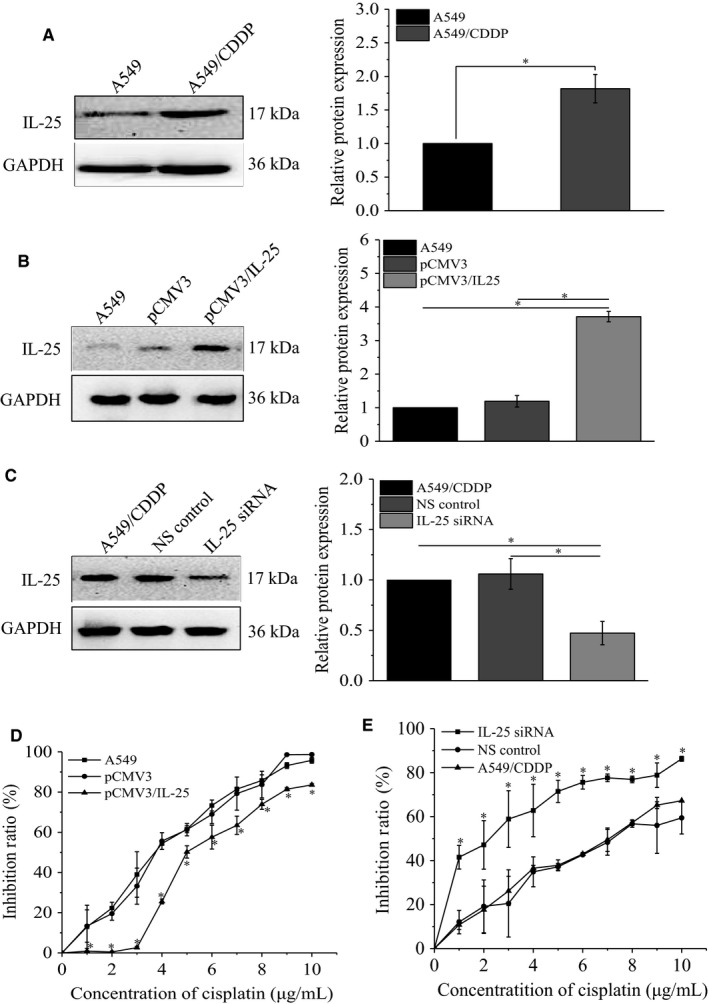
The expression of IL‐25 affects the cisplatin resistance of lung cancer cells. A, The expression of IL‐25 in A549 and A549/CDDP cells. B, IL‐25 overexpressed in A549 cells. C, silence of IL‐25 expression by siRNA in A549/CDDP cells. D, IL‐25 overexpression desensitizes A549 cells to cisplatin treatment. E, Silence of IL‐25 expression sensitizes A549/CDDP cells to cisplatin treatment. *P < 0.01 vs indicated controls, all figures represent the average of three sets of independent experiments

In order to investigate whether IL‐25 expression might be involved in the formation of cisplatin resistance in A549 cells, we first detected the IL‐25 expression by western blotting assay in A549 and A549/CDDP cells. Our data showed that, compared to A549 cells, A549/CDDP cells enhanced the expression of IL‐25 (Figure 2A). Then, we successfully overexpressed IL‐25 in A549 cells and silenced IL‐25 expression in A549/CDDP cells by transfecting the overexpression plasmid or knockdown siRNA, respectively (Figure 2B,C). To further investigate whether alteration of IL‐25 expression might affect the cisplatin resistance in A549 cells, CCK‐8 assay was used to study the effect of IL‐25 expression on the cisplatin tolerance in these cells. Our results indicated that IL‐25 overexpressed A549 cells showed greater resistant abilities to the cisplatin treatment, compared to that in A549 cells and A549 cells transfected with pCMV3 control plasmid (Figure 2D). Whereas, IL‐25 silenced A549/CDDP cells exhibited frailer resistant abilities to the cisplatin treatment, compared to that in A549/CDDP cells and A549/CDDP cells transfected with NS control siRNA (Figure 2E). These data suggested that IL‐25 is involved in cisplatin resistance in lung cancer cells.

### 3.3 Extracellular secreted IL‐25 desensitizes lung cancer cells to cisplatin treatment

**Figure 3:**
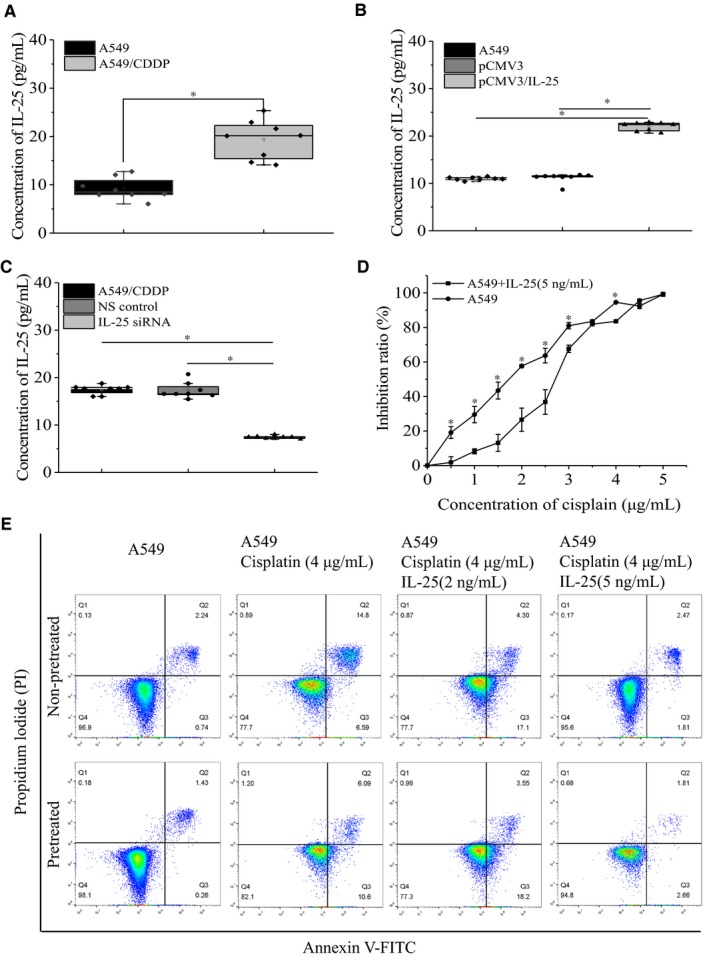
Extracellular IL‐25 affects the cisplatin resistance of lung cancer cells. ELISA assay the expression of IL‐25 in the supernatant of A549 and A549/CDDP cells (A), in the supernatant of IL‐25 overexpressed A549 cells (B) and IL‐25 silenced A549/CDDP cells (C). D, Exogenous IL‐25 desensitizes A549 cells to cisplatin treatment. E, Apoptosis assay the effect of exogenous IL‐25 on the sensitivity of A549 cells to cisplatin. *P < 0.01 vs indicated controls, all figures represent the average of three sets of independent experiments

Given that IL‐25 is a cytokine which can be secreted into extracellular matrix, we detected the extracellular IL‐25 protein level by ELISA in A549 and A549/CDDP cells. Our data showed that, compared to A549 cells, A549/CDDP cells enhanced the secretion of IL‐25 (Figure 3A). Additionally, we found that overexpression of IL‐25 in A549 cells also increased its secretion (Figure 3B), while silencing of IL‐25 in A549/CDDP cells decreased its secretion (Figure 3C). To further confirm that extracellularly secreted IL‐25 is responsible for cisplatin resistance in A549 cells, we treated A549 cells with exogenous IL‐25 and then tested the cells’ tolerance to cisplatin. First, A549 cells were subjected to different concentration of IL‐25 for 24 hours to detect the cytotoxicity of IL‐25 on cells. The results of flow cytometry analysis demonstrated that low concentration of exogenous IL‐25 (2 and 5 ng/mL) had no effect on A549 cells, even higher concentration of exogenous IL‐25 (10, 50, and 100 ng/mL) barely caused apoptosis (Figure S1B). Then, 5 ng/mL of exogenous IL‐25 was used to pretreat A549 cells for 24 hours, and the cisplatin‐resistant abilities of pretreated cells were measured. Our results showed that, compared with untreated A549 cells, A549 cells treated with IL‐25 had greater resistant capabilities (Figure 3D). Moreover, we found that whether treated with IL‐25 in advance or not the exogenous IL‐25 could significantly reduce the sensitivity of A549 cells to cisplatin (Figure 3E). Together, these data suggested that extracellular secreted IL‐25 promotes the formation of cisplatin resistance in lung cancer cells.

### 3.4 Major vault protein is the key factor for IL‐25 mediated cisplatin resistance of lung cancer cells

**Figure 4:**
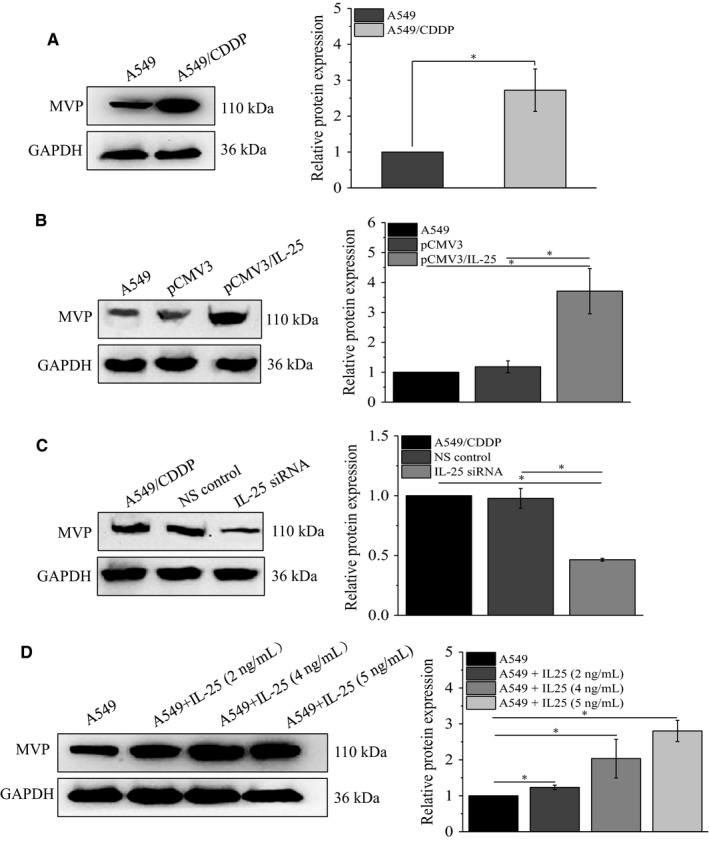
IL‐25 regulates the expression of major vault protein (MVP). A, The expression of MVP in A549 and A549/CDDP cells, in IL‐25 overexpressed A549 cells (B) and in IL‐25 silenced A549/CDDP cells (C). D, The effect of exogenous IL‐25 on the expression of MVP in A549 cells. *P < 0.01 vs indicated controls, all figures represent the average of three sets of independent experiments

To further illustrate how IL‐25 regulates cisplatin resistance of lung cancer cells, we examined the expression of MVP, which is a well‐known multidrug‐resistant protein, in cisplatin‐resistant A549/CDDP cells. We found that, comparing with in A549 cells, the protein level of MVP was significantly elevated in A549/CDDP cells (Figure 4A). Additionally, we examined the effect of altering IL‐25 expression on the expression of MVP. Our data showed that overexpression of IL‐25 increased the MVP expression in A549 cells (Figure 4B), while knockdown of IL‐25 decreased the MVP expression in A549/CDDP cells (Figure 4C). Moreover, the exogenous IL‐25 also significantly induced the expression of MVP in A549 cells (Figure 4D). Interestingly, we also observed an IL‐25 concentration‐dependent increase of Bcl‐2 which is a well‐known anti‐apoptosis marker, while the apoptosis executive factor cleaved caspase3 has no significant change (Figure S2A). These data indicated that IL‐25 might enhance cisplatin resistance in lung cancer cells at least partly by increasing the expression of MVP and by enhancing its antiapoptotic ability.

### 3.5 IL‐25‐induced MVP elevation in lung cancer cells requires NF‐κB activity

**Figure 5:**
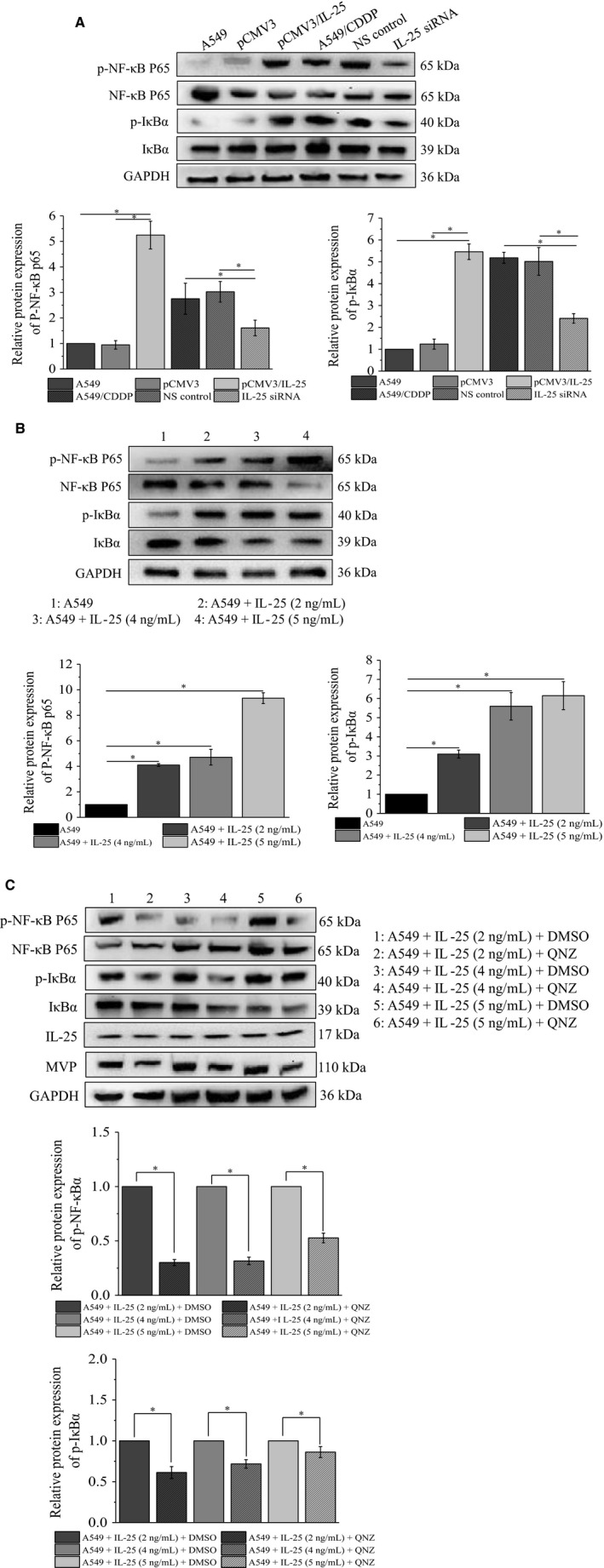
IL‐25 regulates the NF‐κB signaling pathway activity. A, The change of NF‐κB signaling pathway in IL‐25 overexpressed A549 cells and IL‐25 silenced A549/CDDP cells. B, The effect of exogenous IL‐25 on the activity of NF‐κB signaling pathway in A549 cells. C, QNZ blocks the effect of exogenous IL‐25 on the expression of major vault protein (MVP) and the activity of NF‐κB signaling pathway in A549 cells. *P < 0.01 vs indicated controls, all figures represent the average of three sets of independent experiments

IL‐25 is an NF‐κB signaling pathway activator participating in multiple physiological processes of tumor cell, such as proliferation, apoptosis, and metastasis.^32^, ^33^ Therefore, we supposed that IL‐25 mediates MVP expression by activating NF‐κB signaling pathway. To confirm our hypothesis, we detected the expression of NF‐κB signaling pathway‐associated proteins by western blot in IL‐25 overexpressed and silenced cells. Our data showed that overexpression of IL‐25 increased the phosphorylation of NF‐κB P65 and IκBα in A549 cells, while knockdown of IL‐25 decreased the phosphorylation of NF‐κB P65 and IκBα in A549/CDDP cells (Figure 5A). Meanwhile, our data also showed that exogenous IL‐25 increased the phosphorylation of NF‐κB P65 and IκBα in a concentration‐dependent manner (Figure 5B). To further illustrate that the NF‐κB activity is involved in IL‐25 mediated the regulation of MVP expression, we used QNZ (a NF‐κB inhibitor, 11 nmoL/mL) simultaneously with IL‐25 treating A549 cells, and the related proteins were detected by western blot at 24 hours after the treatment. Our data demonstrated, comparing with each DMSO control group, QNZ did not affect the expression of IL‐25 but inhibit the IL‐25‐induced phosphorylation of NF‐κB P65 and IκBα as well as the expression of MVP (Figure 5C). These data indicated that the IL‐25‐induced expression of MVP is dependent on the activation of NF‐κB signaling pathway.

### 3.6 Exogenous IL‐25 enhances cisplatin tolerance of lung cancer tumors, in vivo

**Figure 6:**
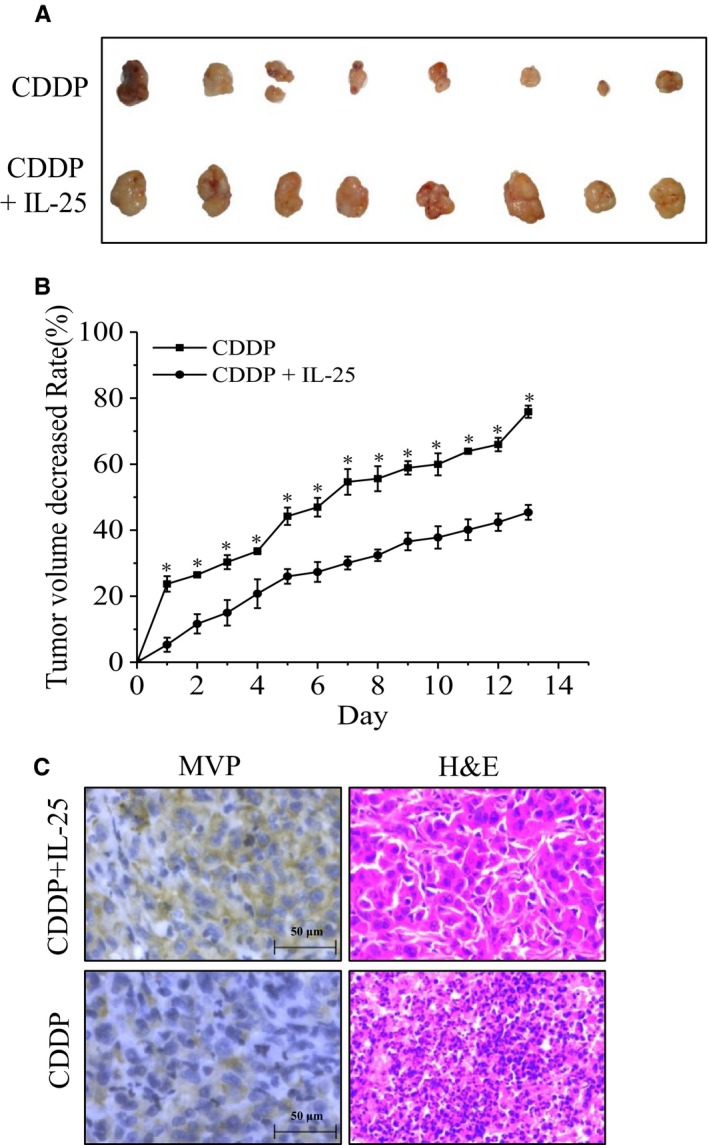
The effect of IL‐25 in vivo experiment. A, The tumor sizes of cisplatin alone treated nude mice and cisplatin along with IL‐25 treated nude mice (n = 8). B, The average reduced rates of tumor volume in indicated groups. C, Representative microphotographs showing immunohistochemical staining analysis the expression of major vault protein (MVP) (magnification, ×100)

The role of IL‐25 in cisplatin tolerance of lung cancer cells was also investigated in animal model. After a 2‐week treatment, the tumor sizes of cisplatin (5 mg/kg) alone treated nude mice (n = 8) were smaller than that in cisplatin along with IL‐25 (5 ng/mL) treated nude mice (Figure 6A). The average reduced rates of tumor volume were significantly lower in IL‐25 and cisplatin treated group than that in cisplatin treated control group (Figure 6B). Immunohistochemical staining analysis revealed extensive expression of MVP in the tumor tissues from IL‐25 and cisplatin treated group, whereas MVP expression was barely detected in the tumor tissues from only cisplatin treated group; meanwhile, the number of inflammatory cells were significantly reduced in the cisplatin and IL‐25 treated tumors (Figure 6C). The IL‐25 induced cisplatin tolerance was also verified by the elevated expression of Bcl‐2 and the reduced expression of cleaved caspase3 (Figure S2B). These data suggested that the existence of extracellular IL‐25 enhanced the cisplatin resistance of A549.

## 4 DISCUSSION

Acquired drug resistance in lung cancer cells is the main reason for the failure of chemotherapy treatment. There are many reasons for the drug resistance of lung cancer cells, but the molecular mechanism is not fully explained yet. In this study, we constructed a drug‐resistant lung adenocarcinoma cell line A549/CDDP. Consistent with our previously report, the IC_50_ of A549/CDDP cells was significantly enhanced.^34^ Interestingly, we found that the IC_50_ of parental A549 cells which we used in this study was significantly higher than that reported in our previous study, while the IC_50_ of A549/CDDP cell lines was lower than that reported in previously study.^34^ We suspect that it may be the parental A549 cells accumulating resistance‐related mutations during continuous passage, such as ROS mutations, resulting in differences in drug sensitivity of the same cell line. The genome‐wide sequencing analysis of two parental A549 cells is currently underway.

IL‐25 may play different roles in different cancers, either as a cancer suppressor or as a cancer promoter. In this study, we found that IL‐25 is highly expressed in cisplatin‐resistant cells. Manipulating the expression of IL‐25 in A549 cells or A549/CDDP cells changed their resistance to cisplatin accordingly. In addition, we also noticed that A549/CDDP cells increased the amount of extracellular IL‐25, and A549 cells treated with exogenous IL‐25 also enhanced its tolerance to cisplatin. It was also reported that, in the early stage, IL‐25 had a killing effect on lung cancer cells.^13^ However, even when treating the A549 cells with a high concentration of IL‐25 (600 ng/mL), no obvious apoptosis was observed (data not shown). Conversely, by treatment with IL‐25 (5 ng/mL), A549 cells had significantly higher tumorigenic capacity in vivo than the nontreated A549 cells (data not shown). Our data suggest that IL‐25 plays critical roles in promoting drug‐resistance of lung cancer cells.

Further study revealed that IL‐25 regulates cisplatin resistance of lung cancer cells by mediating the expression of MVP. Either intracellular overexpression of IL‐25 or exogenously treated with IL‐25 in A549 cells could increase the expression of MVP and enhance the tolerance to cisplatin. However, the molecular mechanism is not explained clearly. Previous studies have reported that IL‐25 activates the NF‐κB pathway to mediate inflammatory response,^35^, ^36^ and the activated NF‐κB could inhibit the occurrence of apoptosis.^37^, ^38^, ^39^ Therefore, next we explored whether NF‐κB signaling pathway is involved in the regulation of MVP expression. We found that IL‐25, especially extracellular IL‐25, could regulate the activity of NF‐κB signaling pathway and affect the expression of MVP. Moreover, we also found the expression of Bcl‐2 in exogenous IL‐25 treated A549 cells increased in a concentration‐dependent manner. In IL‐25 treated In vivo experiments group, we detected the elevated expression of Bcl‐2. Together, these findings suggest, in addition to inducting anti‐apoptosis, IL‐25 promotes cisplatin resistance of lung cancer cells partly by activating NF‐κB signaling pathway to increase the expression of MVP.

In conclusion, our findings revealed a novel mechanism of IL‐25‐ NF‐κB‐MVP cascade regulated formation of drug resistance. By targeting IL‐25 or its receptor may provide a potential therapy strategy for reversing cisplatin resistance of human lung adenocarcinoma.

## CONFLICT OF INTEREST

The authors of this article declared they have no conflicts of interest.
