# Supplementary material for: IL‐25 promotes cisplatin resistance of lung cancer cells by activating NF‐κB signaling pathway to increase of major vault protein
Source: Cancer Med. 2019 May 1;8(7):3491–501. doi: 10.1002/cam4.2213 (PMC6601590; doi:10.1002/cam4.2213)
Supplement: Supplementary file 1 [file CAM4-8-3491-s001.pdf]

Supplementary Figure 1

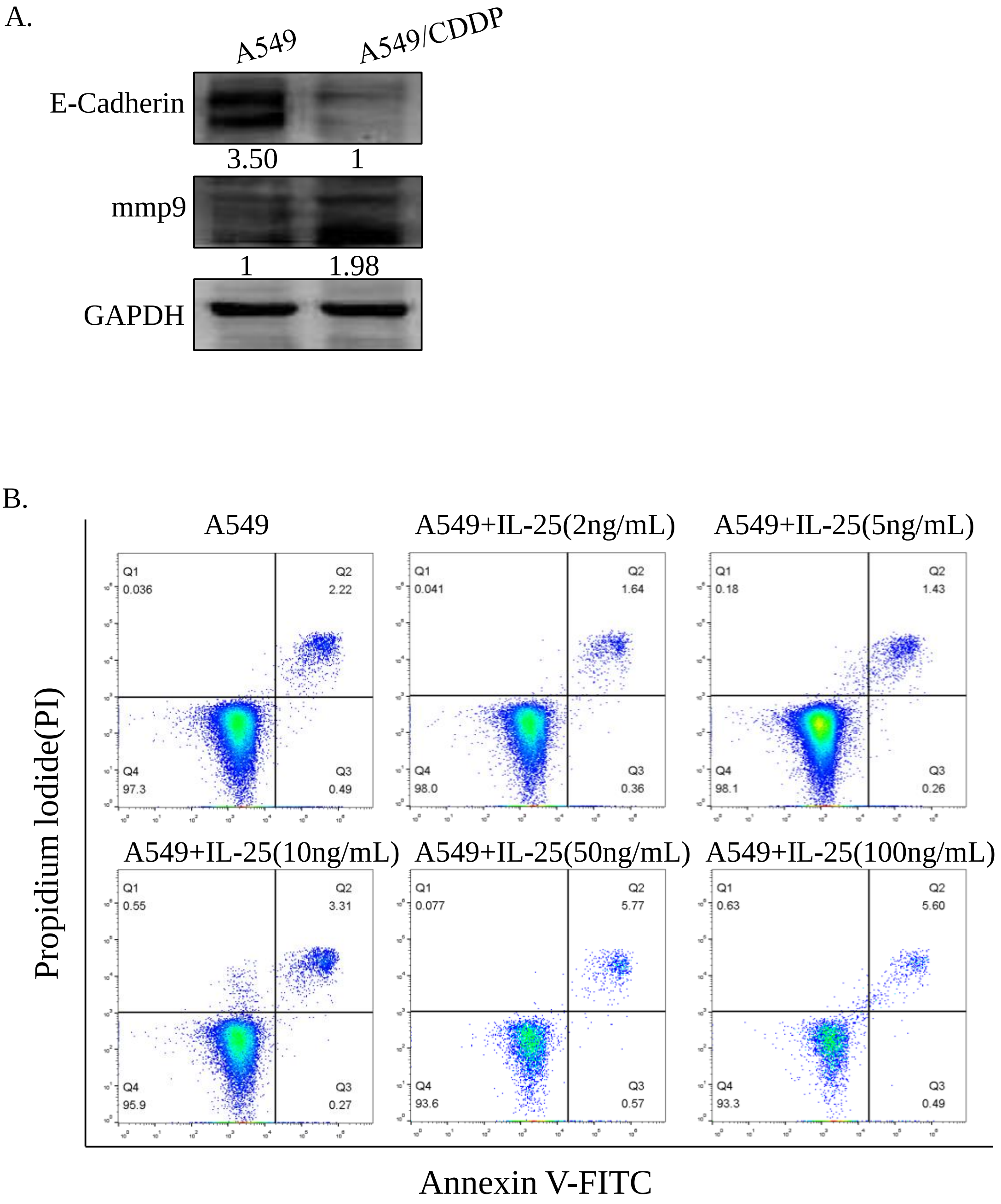

Figure S1. (A) the expression of E-cadherin and mmp9 in A549/CDDP cells, parental A549 cells served as control. (B) Flowcytometry analysis the apoptosis ratio in different concentration of exogenous IL-25 treated A549 cells.

Supplementary Figure 2

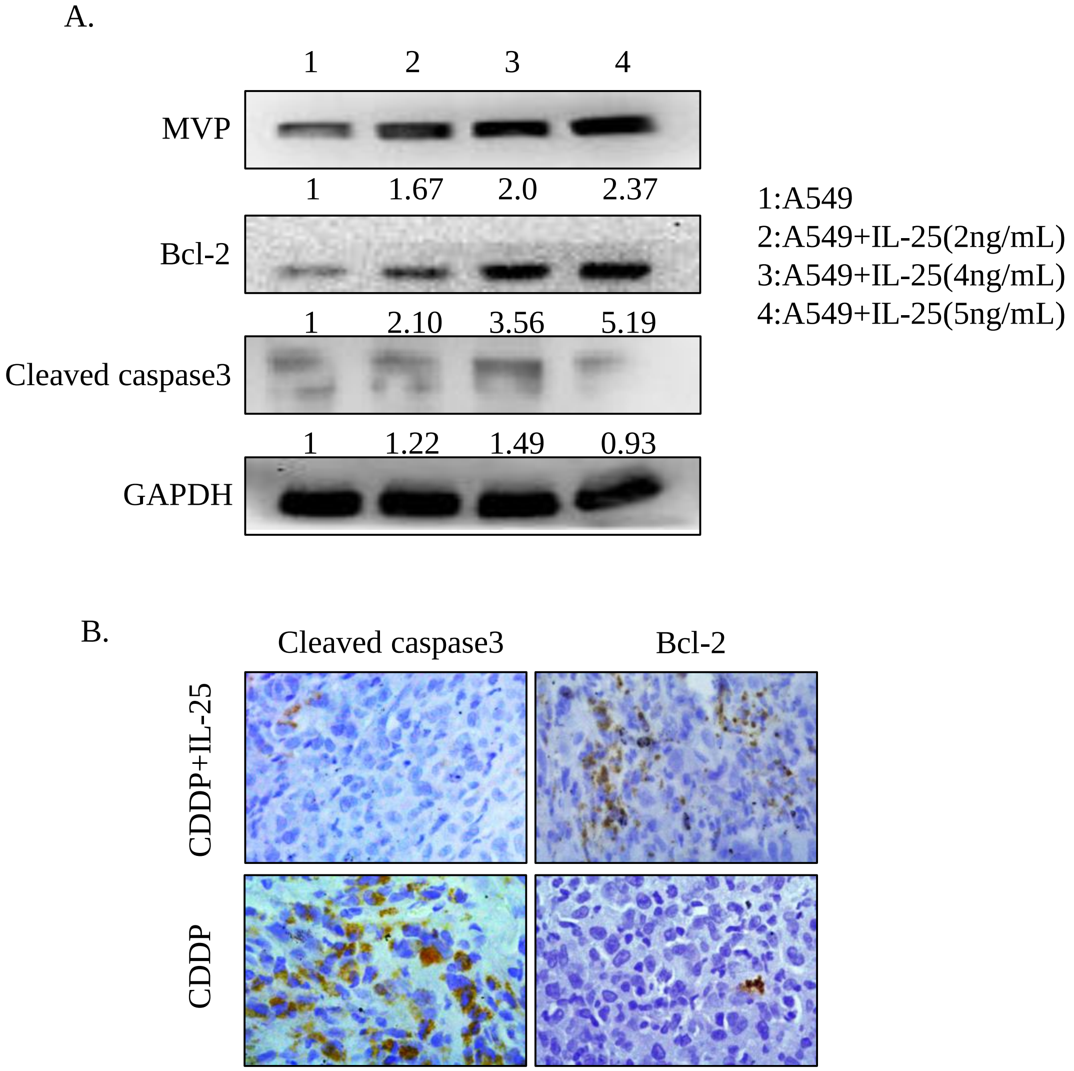

Figure S2. (A) The expression of Bcl-2 and cleaved caspase 3 in IL-25 treated A549 cells. (B) Representative microphotographs showing immunohistochemical staining analysis the expression of Bcl-2 and cleaved caspase 3 in tumor tissues (magnification, × 100).
